# Interleukin-1β mediates metalloproteinase-dependent renal cell carcinoma tumor cell invasion through the activation of CCAAT enhancer binding protein β

**DOI:** 10.1002/cam4.7

**Published:** 2012-06-07

**Authors:** Brenda L Petrella, Matthew P Vincenti

**Affiliations:** 1 Research Service, Department of Veterans Affairs, VA Medical CenterWhite River Junction, Vermont; 2 Department of Medicine, Geisel School of Medicine at DartmouthLebanon, New Hampshire

**Keywords:** CCAAT enhancer binding protein β, interleukin-1β, matrix metalloproteinases, renal cell carcinoma, tumor invasion

## Abstract

Effective treatment of metastatic renal cell carcinoma (RCC) remains a major medical concern, as these tumors are refractory to standard therapies and prognosis is poor. Although molecularly targeted therapies have shown some promise in the treatment of this disease, advanced RCC tumors often develop resistance to these drugs. Dissecting the molecular mechanisms underlying the progression to advanced disease is necessary to design alternative and improved treatment strategies. Tumor-associated macrophages (TAMs) found in aggressive RCC tumors produce a variety of inflammatory cytokines, including interleukin-1β (IL-1β). Moreover, the presence of TAMs and high serum levels of IL-1β in RCC patients correlate with advanced disease. We hypothesized that IL-1β in the tumor microenvironment promotes the development of aggressive RCC tumors by directing affecting tumor epithelial cells. To address this, we investigated the role of IL-1β in mediating RCC tumor cell invasion as a measure of tumor progression. We report that IL-1β induced tumor cell invasion of RCC cells through a process that was dependent on the activity of matrix metalloproteinases (MMPs) and was independent of migration rate. Specifically, IL-1β induced the expression of MMP-1, MMP-3, MMP-10, and MT1-MMP in a mechanism dependent on IL-1β activation of the transcription factor CCAAT enhancer binding protein β (CEBPβ). Consistent with its role in MMP gene expression, CEBPβ knockdown significantly reduced invasion, but not migration, of RCC tumor cells. These results identify the IL-1β /CEBPβ/MMP pathway as a putative target in the design of anti-metastatic therapies for the treatment of advanced RCC.

## Introduction

For 2012, it is predicted that nearly 65,000 new cases of cancer of the kidney and renal pelvis will be diagnosed, and approximately 13,500 of those patients will die from disease-specific causes [1]. Renal cell carcinoma (RCC) is the most common urological cancer and represents the seventh most common cancer in men and the ninth most common cancer in women. When diagnosed and treated early, localized RCC has an excellent prognosis, with 90% of patients surviving beyond 5 years from diagnosis [2]. However, many RCC patients are asymptomatic, and the malignancy may go undetected for some time. This delay in diagnosis is reflected by the observation that approximately 30% of RCC patients present with metastatic disease at the time of diagnosis [3]. Prognosis for patients with metastatic disease is poor compared to patients with localized cancer, with the 5-year survival rate dropping to 11% for metastatic patients [2]. In fact, the majority of RCC patients with metastatic disease will not survive 6 months beyond diagnosis. Therefore, understanding the molecular pathways involved in RCC metastasis will identify novel targets for the treatment of this disease.

Clear cell RCC, the most common form of kidney cancer, is genetically linked to the von Hippel Lindau (VHL) tumor suppressor gene in familial and sporadic cases [4]. VHL functions normally to destabilize hypoxia-inducible factor alpha (HIF-α) protein, a key transcription factor that drives renal tumorigenesis. Dysregulated expression of HIF-α as a direct consequence of VHL inactivation results in tumors that display a hypoxic phenotype, even when the tumor microenvironment is not in fact hypoxic. As a result, clear cell RCC tumors overexpress proangiogenic factors, such as vascular endothelial growth factor (VEGF), and are highly vascularized [4]. It is well accepted that tumor angiogenesis is directed, in part, by inflammatory mediators in the microenvironment, such as those expressed by tumor-associated macrophages (TAMs) [5]. In particular, the proinflammatory cytokine, interleukin-1β (IL-1β), has been shown to be important in promoting tumor angiogenesis. Studies in mouse models have demonstrated that inhibition of IL-1β signaling, although IL-1 receptor antagonists or knockout of the IL-1β gene, reduced tumor blood vessel formation [6]. TAMs isolated from RCC tumors express high levels of inflammatory cytokines, including IL-1β, tumor necrosis factor α (TNFα), and interleukin-6 (IL-6; [7]). The presence of TAMs and high serum levels of these cytokines are poor prognostic factors in RCC patients, indicating that an inflammatory microenvironment may promote RCC tumor progression [8–10].

In addition to its role in angiogenesis, we hypothesized that IL-1β contributes to the progression of RCC by directly affecting tumor cell invasion. Our results show that IL-1β potently induced the invasiveness of VHL null RCC cells, a process that required the activity of the matrix metalloproteinase (MMP) family of endopeptidases. Collectively, the MMPs degrade every component of the extracellular matrix (ECM) as well as nonmatrix proteins, such as growth factors, adhesion molecules, and other MMPs. Overexpression of MMPs is associated with the progression of many human cancers [11]. We demonstrate that IL-1β induced the expression of the secreted MMPs: MMP-1 (Collagenase-1), MMP-3 (Stromelysin-1), MMP-10 (Stromelysin-2), and the transmembrane MMP, MT1-MMP (MMP-14), in RCC cells. In addition, the mechanism of IL-1β-induced MMP expression was mediated by CCAAT enhancer binding protein β (CEBPβ). CEBPβ is a basic leucine zipper (bZIP) transcription factor shown to contribute to the pathogenesis of breast cancer [12]. Consistent with this, our data show that IL-1β-induced RCC tumor invasion was dependent on CEBPβ. We conclude from this study that the IL-1β/CEBPβ/MMP pathway is a major mediator of RCC tumor invasion and should be considered in the design of molecularly targeted therapeutics.

## Material and Methods

### Cell culture and treatments

The human 786-0 RCC cell line (American Type Culture Collection, Manassas, VA) is null for VHL expression [13]. Cells were maintained in Dulbecco's Modified Eagle's Medium (Mediatech, Inc., Herndon, VA) supplemented with 10% fetal bovine serum (FBS; Hyclone, Logan, UT), penicillin (100 U/mL) and streptomycin (100 g/mL), and cultured at 37°C, 5% CO_2_. For serum-free conditions, culture medium without supplementation of FBS was used. Cells were washed three times with Hank's Balanced Salt Solution (HBSS; Mediatech, Inc.) prior to treatment in serum-free conditions. IL-1β (R&D Systems, Minneapolis, MN) was used at the indicated concentrations and times. GM6001 (Millipore, Inc., Bellerica, MA), was prepared in DMSO and used at 25 μM. An equal volume of DMSO was used as a vehicle control.

### Stable transfection

The 786-0 cells were transfected to stably express SA Biosciences (Valencia, CA) SureSilencing CEBPβ shRNA (Cat no. KH00991N) or a control scrambled shRNA using Lipofectamine 2000™ Transfection Reagent (Invitrogen, Carlsbad, CA) following manufacturer's instructions. Cells were allowed to recover in fresh DMEM + 10% FBS for 24 h before selection. Transfected cells were diluted 1:1000 and selected with culturing media containing 1 μg/mL G418 for approximately 2 weeks. Individual colonies were picked and expanded for analysis and cultured under continuous selection. CEBPβ knockdown in the polyclonal population and in the individual clones was assessed through Western blotting of whole cell extracts. Scrambled shRNA clones were selected for further analysis if they expressed comparable levels of CEBPβ as the 786-0 parental cells. CEBPβ shRNA-expressing cells displaying >50% knockdown compared to scrambled shRNA control cells were used in experiments.

### Immunoblotting

Whole cell lysates were harvested from confluent 6-well with 2× sample buffer (Sigma Chemical, St. Louis, MO). Secreted proteins were isolated from conditioned media using trichloroacetic acid (TCA) precipitation. Samples were resolved by SDS-PAGE as previously described [14]. Blots were probed with antibodies to total CEBPβ (Santa Cruz Biotechnology, Santa Cruz, CA), phospho-CEBPβ (Thr235), and actin (Cell Signaling Technology, Beverly, MA), MMP-1 (Millipore, Inc.), MMP-3 (R&D Systems), MMP-10 (R&D Systems), and MT1-MMP (Millipore, Inc.). Proteins were visualized using the ThermoPierce Biotechnology (Rockford, IL) Supersignal West Pico or Femto detection kits.

### Immunofluorescence

The 786-0 cells were seeded at a density of 2 × 10^4^ cells/well of an 8-well Lab-Tek Chambered #1.0 Borosilicate Coverglass System (Thermo Fisher Scientific, Waltham, MA). Cells were treated overnight in serum-free media in the presence or absence of 1 ng/mL IL-1β. The following day, cells were fixed with 4% paraformaldehyde at room temperature for 15 min. Antigens were blocked at room temperature for 30 min with a blocking buffer containing 1× PBS, 10% normal goat serum, 1% BSA, and 0.3% Triton X-100. Cells were washed with 1× PBS + 1% BSA between each step. The human EMT 3-color immunocytochemistry kit (R&D Systems) was used for analysis of EMT markers, Vimentin, E-Cadherin and Snail, according to the manufacturer's instructions. For analysis of CEBPβ expression, blocked cells were incubated at room temperature for 2 h with anti-CEBPβ antibody (sc-150, Santa Cruz Biotechnology) diluted 1:100 in blocking buffer. No primary antibody was added in control wells to account for background fluorescence. Goat anti-rabbit Alexa 488 secondary antibody (Invitrogen) was added in blocking buffer at (10 μg/mL) and incubated for an additional hour at room temperature in the dark. Before viewing, cells were thrice washed with 1× PBS + 1% BSA and ProLong® Gold Antifade Reagent (Invitrogen) was added as a DAPI nuclear counterstain. Immunofluorescence was viewed using a Nikon 90i inverted phase contrast microscope using 20× and 40× objectives, as indicated, and analyzed using NIS Elements Advanced Research software.

### Real-time RT-PCR

Purification of total cellular RNA, reverse transcription, and real-time PCR reactions were all performed as described previously [14]. Briefly, for each experiment, triplicate samples were obtained, and the cDNA for each was assayed in duplicate. A quantity of 50 ng of input cDNA was used in each real-time PCR reaction, and amplification was measured using the following parameters: 95°C 10 min, 40 cycles of 95°C 15 sec, 60°C 1 min, and a plate read. Primer sequences were as published previously [15, 16]. Data are presented as relative expression as calculated using 2^−ΔΔC(T)^ method [17].

### Migration and collagen invasion assays

HTS FluoroBlok™ inserts (BD Falcon Labware, 6.5 mm, Franklin Lakes, NJ) were used for both migration and invasion assays. Migration assays were performed as described below without the inclusion of a collagen matrix. Invasion assays were essentially performed as previously described with a few modifications [18]. Briefly, collagen was prepared from purified bovine type I collagen (Trevigen, Gaithersburg, MD) following manufacturer's instructions. Collagen was neutralized with the addition of 10× PBS and 7.5% sodium bicarbonate. FluoroBlok™ membranes were coated with 100 μL per cm^2^ growth area with collagen diluted to 1 mg/mL. The collagen was allowed to gel on top of the transwell membranes at 37°C overnight. Cells for migration or invasion assays were serum-starved overnight with or without treatment, as indicated, before being harvested for assays. The next day, cells were seeded on top of the collagen layer (for invasion assays) or on top of the Fluoroblok™ membrane (for migration assays) at a density of 2 × 10^4^ cells per insert in serum-free media containing 1 ng/mL IL-1β or no treatment. DMEM + 10% FBS was added to the lower chamber as a chemoattractant, and cells were allowed to invade or migrate for 24 h. Invaded cells were stained with Calcein AM (4 μM, Invitrogen) and viewed using fluorescence microscopy using a Nikon Eclipse TS100 inverted microscope equipped with Spot digital software. Micrographs were taken using a 2× objective. Images were imported into Image J and converted to a threshold image for analysis. Cell numbers were derived from threshold counts of the outlines of cells with a circularity of 0.00–1.00 and an area size exclusion of <15 (pixel^2^). Each condition was run in triplicate and repeated at least three times. Data are presented as average fold induction of untreated samples derived from the average threshold count of triplicate samples.

### FITC-collagen degradation assay

786-0 cells were treated overnight in serum-free media alone or supplemented with 1 ng/mL IL-1β. The following day, cells were harvested by trypsinization, and 1 × 10^5^ cells were resuspended in 1 mL serum-free media in the presence or absence of IL-1β. An equal volume of cells (250 μL) was added to an equal volume (250 μL) of a collagen mixture and applied to a 24-well plate (*n* = 3) for a final collagen concentration of 1 mg/mL. The collagen was prepared as a 2 mg/mL mixture of purified bovine type I collagen (Trevigen) and a bovine type I FITC-collagen conjugate (Sigma-Aldrich, St. Louis, MO). Collagen was neutralized with the addition of 10× PBS and 7.5% sodium bicarbonate. The cell-embedded collagen was allowed to solidify overnight at 37°C before 500 μL of prewarmed serum-free media (±IL-1β @ 1 ng/mL) was added to the top of the gel. The following day, 100 μL of media was transferred, and fluorescence of the media was measured at 490/520.

### Statistical analysis

Statistical significance was calculated using the student's *t*-test available at http://www.physics.csbsju.edu/stats/t-test.html and is represented as ± standard deviation (SD) of the mean. Significance was assigned to *P*-values <0.05.

## Results

### IL-1β induces RCC tumor cell invasion

**Figure 1:**
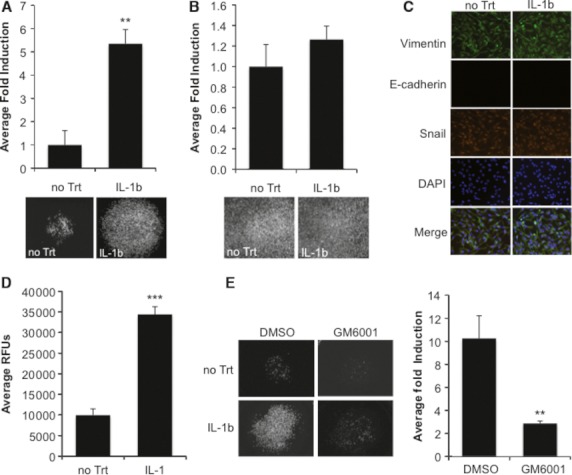
IL-1β induces collagen invasion and degradation. (A) 786-0 RCC cells were treated overnight in serum-free media either alone or containing 1 ng/mL IL-1β. The next day, cells were harvested for an invasion assay as described in the Methods and were allowed to invade overnight. Values are presented as average fold induction of untreated cells, error bars are standard deviation of the mean; *P* < 0.005. (B) 786-0 RCC cells were treated overnight in serum-free media alone or containing 1 ng/mL IL-1β. The next day, cells were harvested for a migration assay as described in the Methods and were allowed to migrate overnight. Values are presented as average fold induction of untreated cells. (C) 786-0 RCC cells were seeded onto glass chamber slides and were untreated or treated with 1 ng/mL IL-1β in serum-free media overnight. Cells were fixed in 4% paraformaldehyde and incubated with fluorochrome-conjugated antibodies against Vimentin (green), E-Cadherin (red), and Snail (orange). Cells were counter-stained with ProLong® Gold Antifade Reagent (DAPI, blue). (D) 786-0 RCC cells were untreated or treated with 1 ng/mL IL-1β in serum-free media overnight. The next day, cells were harvested and subjected to the FITC collagen degradation assay as described in the Methods. Values represent average relative fluorescence units of triplicate samples; *P* < 0.0001. (E) 786-0 RCC cells were treated overnight in serum-free media either alone or containing 1 ng/mL IL-1β. The next day, cells were harvested for an invasion assay as described in the Methods with the following treatments: (1) no treatment + DMSO, (2) no treatment + 25 μM GM6001, (3) 1 ng/mL IL-1β + DMSO or (4) 1 ng/mL IL-1β + 25 μM GM6001. Cells were allowed to invade for 24 h. Values are presented as average fold induction of untreated cells, error bars are standard deviation of the mean; *P* < 0.005.

To begin our investigation of whether the inflammatory cytokine, IL-1β, promoted an invasive phenotype in RCC, we first measured whether or not IL-1β treatment induced collagen invasion of the human 786-0 VHL null RCC cell line. The tumor stromal microenvironment is primarily comprised of type I collagen; therefore, tumor cells must breach this matrix barrier to invade the surrounding tissue [19]. Furthermore, type I collagen is the major protein constituent of bone. Procollagen type I amino-terminal propeptide, a marker of type I collagen metabolism, is significantly elevated in RCC patients with bone metastases [20]. Thus, RCC degradation and invasion of type I collagen has important clinical implications. The 786-0 cells were serum-starved overnight in the presence or absence of IL-1β before being seeded on top of type I collagen-coated transwells in the presence or absence of IL-1β stimulation. Treatment of the RCC cells with IL-1β resulted in induction of tumor cell invasion by 24 h (Fig. 1A).

Tumor cell invasion is a multi-step process beginning with the ability of a cell to individually migrate and to modify the ECM [21]. We next tested whether or not IL-1β stimulation affected tumor cell migration using a transwell migration assay lacking a collagen matrix substrate. The 786-0 cells were serum-starved overnight in the presence or absence of IL-1β before being harvested for the migration assay. IL-1β had no effect on the migration of the 786-0 cells, which displayed high levels of basal migration at 24 h (Fig. 1B). In agreement, IL-1β treatment had no effect on the expression of classic epithelial–mesenchymal transition (EMT) markers, as this cell line already displayed an EMT signature, determined by the expression of vimentin and nuclear Snail and the loss of expression of E-cadherin (Fig. 1C; [22]). These results are consistent with a report that activation of the VHL-HIF pathway results in loss of E-cadherin expression, suggesting that VHL null RCC cells undergo EMT at an early stage in tumorigenesis [23].

Next, the ability of the RCC cells to modify a type I collagen matrix in response to IL-1β treatment was assessed. IL-1β stimulated 786-0 cells were embedded within a semi-solid collagen matrix impregnated with a FITC-collagen conjugate. After 24 h in the presence or absence of IL-1β treatment, cleaved FITC-collagen released into the overlying media was quantified. IL-1β-induced type I collagen degradation (Fig. 1D) by the RCC cells, suggesting that IL-1β-induced cell invasion is mediated by cleavage of the collagen substrate. Because type I collagen degradation requires the enzymatic activity of the MMPs [19], we tested whether or not IL-1β-induced tumor invasion was MMP-dependent. Treatment of the RCC cells with GM6001, a pan-MMP inhibitor, blocked IL-1β-induced invasion (Fig. 1E), demonstrating a role for MMPs in this process.

### IL-1β potently induces MMP expression in RCC cells

**Figure 2:**
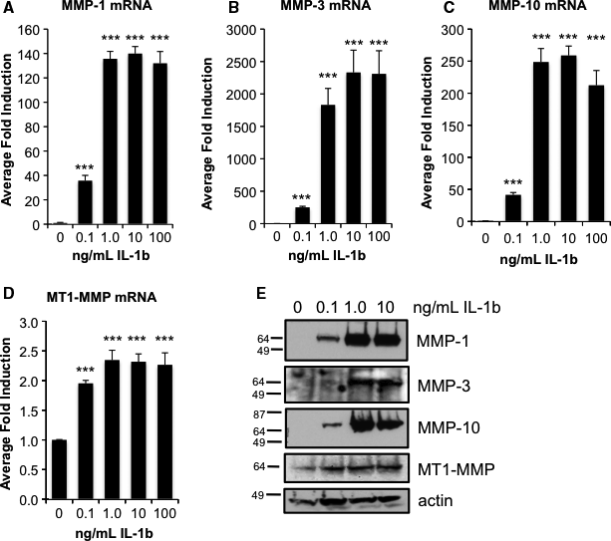
IL-1β induces MMP expression in a dose-responsive manner in RCC cells. 786-0 RCC cells were treated overnight in serum-free media in the presence or absence of 0.1–100 ng/mL IL-1β. The following day, total RNA was harvested, and gene expression was analyzed by real-time PCR for MMP-1 (A), MMP-3 (B), MMP-10 (C), and MT1-MMP (D). Data represent MMP gene expression normalized to GAPDH and calculated as average fold induction of untreated cultures; *P* < 0.0001. (E) 786-0 RCC cells were treated overnight in serum-free media in the presence or absence of 0.1–10 ng/mL IL-1β. The following day, total protein was harvested, and media from the cultures were subjected to TCA precipitation for protein analysis of the secreted MMPs. Protein expression analyses of MMP-1, -3, -10, MT1-MMP, and actin were performed using Western blotting.

Since IL-1β promoted MMP-dependent type I collagen invasion by RCC cells, we next measured the effect of IL-1β on MMP gene and protein expression. Specifically, we focused our analysis on MMPs with type I collagenase activity. Overexpression of MMPs has been associated with several steps in tumorigenesis, such as proliferation, apoptosis, inflammatory response, and tumor invasion and metastasis [11]. The 786-0 cells were treated overnight with 0.1–100 ng/mL IL-1β in serum-free media or serum-free media alone, and total RNA and protein were harvested for analyses. IL-1β potently induced the expression of MMP-1, MMP-3, and MMP-10 at both the mRNA and protein levels in a dose-responsive manner (Fig. 2A–D). MMP-1 collagenase is primarily responsible for cleaving type I collagen found in the tumor stroma. Both MMP-3 and MMP-10 activate MMP-1 and degrade noncollagen ECM proteins [19]. Importantly, MMP-3 and MMP-10 are often coordinately regulated with MMP-1.

MT1-MMP (MMP-14) is a transmembrane protein important for pericellular proteolysis of type I collagen [24]. We previously published that MT1-MMP is regulated by the VHL-HIF pathway and is constitutively expressed in VHL null RCC cells [16], unlike the secreted MMPs (MMP-1, MMP-3, MMP-10), which are not basally expressed in these cells. Constitutive expression of MT1-MMP was mildly induced by IL-1β treatment (Fig. 2D and E). Another collagenase, MMP-13 (collagenase-2), was not expressed in these cells either basally or in response to IL-1β treatment (data not shown). Interestingly, mRNA expression of all four MMPs was maximally induced with 1 ng/mL IL-1β, suggesting that the IL-1β signaling pathway is saturated at this concentration. Therefore, subsequent experiments were conducted using 1 ng/mL IL-1β.

**Figure 3:**
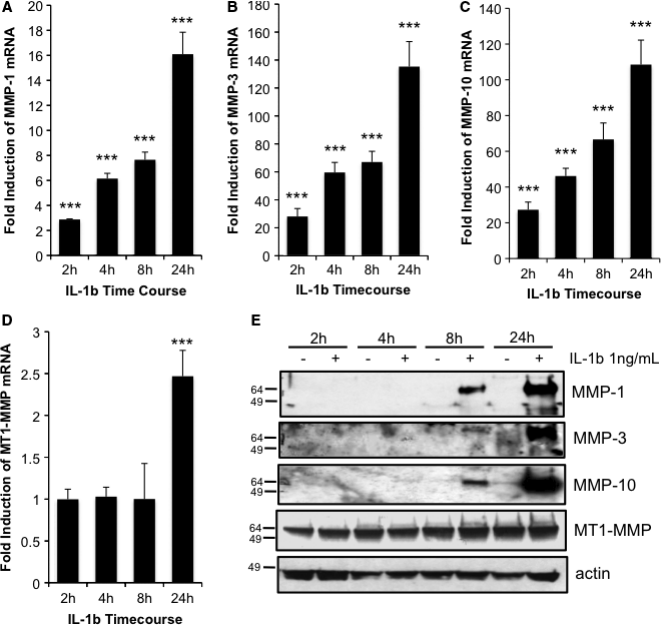
IL-1β induces MMP expression in a time-dependent manner in RCC cells. 786-0 RCC cells were treated overnight in serum-free media in the presence or absence of 1 ng/mL IL-1β. At 2, 4, 8, and 24 h posttreatment, total RNA was harvested, and gene expression was analyzed by real-time PCR for MMP-1 (A), MMP-3 (B), MMP-10 (C), and MT1-MMP (D). Data represent MMP gene expression normalized to GAPDH and calculated as average fold induction of untreated cultures; *P* < 0.0001. (E) 786-0 RCC cells were treated overnight in serum-free media in the presence or absence of 1 ng/mL IL-1β. At 2, 4, 8, and 24 h posttreatment, total protein was harvested, and media from the cultures were subjected to TCA precipitation for protein analysis of the secreted MMPs. Protein expression analyses of MMP-1, -3, -10, MT1-MMP, and actin were performed using Western blotting.

MMP-1, MMP-3, and MMP-10 mRNA expression was induced as early as 2 h by IL-1β and maximally induced by 24 h (Fig. 3A–C). Induced protein levels of MMP-1, MMP-3, and MMP-10 were detected by 8 h and maximum expression was reached by 24 h posttreatment (Fig. 3E). In contrast, MT1-MMP mRNA was not induced until 24 h of IL-1β treatment, and MT1-MMP protein levels remained relatively constant throughout the 24-h timecourse (Fig. 3E).

### IL-1β induces CEBPβ expression in RCC cells

**Figure 4:**
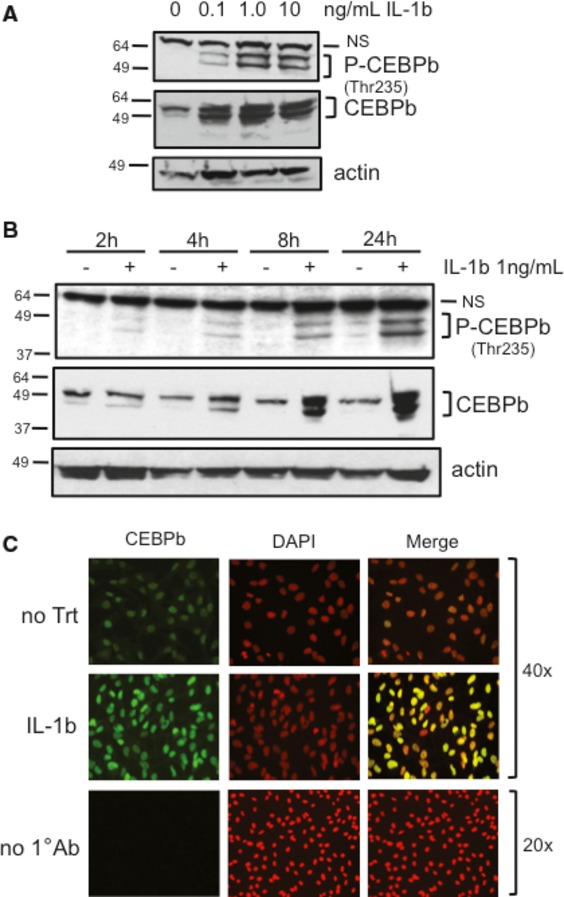
IL-1β induces CEBPβ expression and phosphorylation in RCC cells. (A) 786-0 RCC cells were treated overnight in serum-free media in the presence or absence of 0.1–100 ng/mL IL-1β. The following day, total protein was harvested and immunoblotted for phospho-CEBPβ (Thr235), total CEBPβ, and actin. (B) 786-0 RCC cells were treated overnight in serum-free media in the presence or absence of 1 ng/mL IL-1β. At 2, 4, 8 and 24 h posttreatment, total protein was harvested and immunoblotted for phospho-CEBPβ (Thr235), total CEBPβ, and actin. (C) 786-0 RCC cells were seeded onto glass chamber slides for immunofluorescence analysis of CEBPβ as described in the Methods. The next day, cells were untreated or treated with 1 ng/mL IL-1β in serum-free media overnight before fixation. Immunofluorescent expression of CEBPβ was visualized using Alexa 488-conjugated secondary antibodies (CEBPβ, green). Cells incubated with no primary antibody were included as a negative control for background fluorescence. Cells were counter-stained with ProLong® Gold Antifade Reagent (DAPI, false-colored red). Micrographs were taken using a 20× or 40× objective, as indicated. Merged images of CEBPβ expression and DAPI stain appear yellow.

CEBPβ is a bZIP transcription factor that responds to inflammatory signals [25]. Importantly, CEBPβ activation has been correlated with increased invasiveness of RCC tumors in patients, suggesting it plays a role in the process of tumor cell invasion [26]. Previously, we published that IL-1β induces MMP gene expression in non-small cell lung carcinoma and chondrosarcoma cells, and that IL-1β induction of MMP gene expression in these cancer lines is dependent on the transcriptional activity of the CEBPβ [14, 15]. Therefore, we next investigated whether IL-1β induced the expression and/or activation of CEBPβ. 786-0 cells were treated with 0.1–10 ng/mL IL-1β for 24 h, or at 1 ng/mL in a timecourse experiment (2–24 h), and total protein was isolated for analyses. Similar to the MMP response to IL-1β treatment, total CEBPβ protein was potently induced by IL-1β treatment at 1 ng/mL, and was measurably induced by 2–4 h of IL-1β treatment (Fig. 4A and B). Phosphorylation of CEBPβ at threonine 235 mediates the transcriptional activity of this transcription factor [25]. IL-1β-induced phosphorylation at this site was dose-responsive, and P-CEBPβ was detectable by 2 h posttreatment (Fig. 4A and B). Furthermore, IL-1β-induced CEBPβ was detected in the nuclei of stimulated cells as measured by immunofluorescence (Fig. 4C). Together, these data demonstrate that IL-1β induces CEBPβ expression and activation in RCC cells.

### CEBPβ regulates IL-1β-induced MMP expression in RCC cells

**Figure 5:**
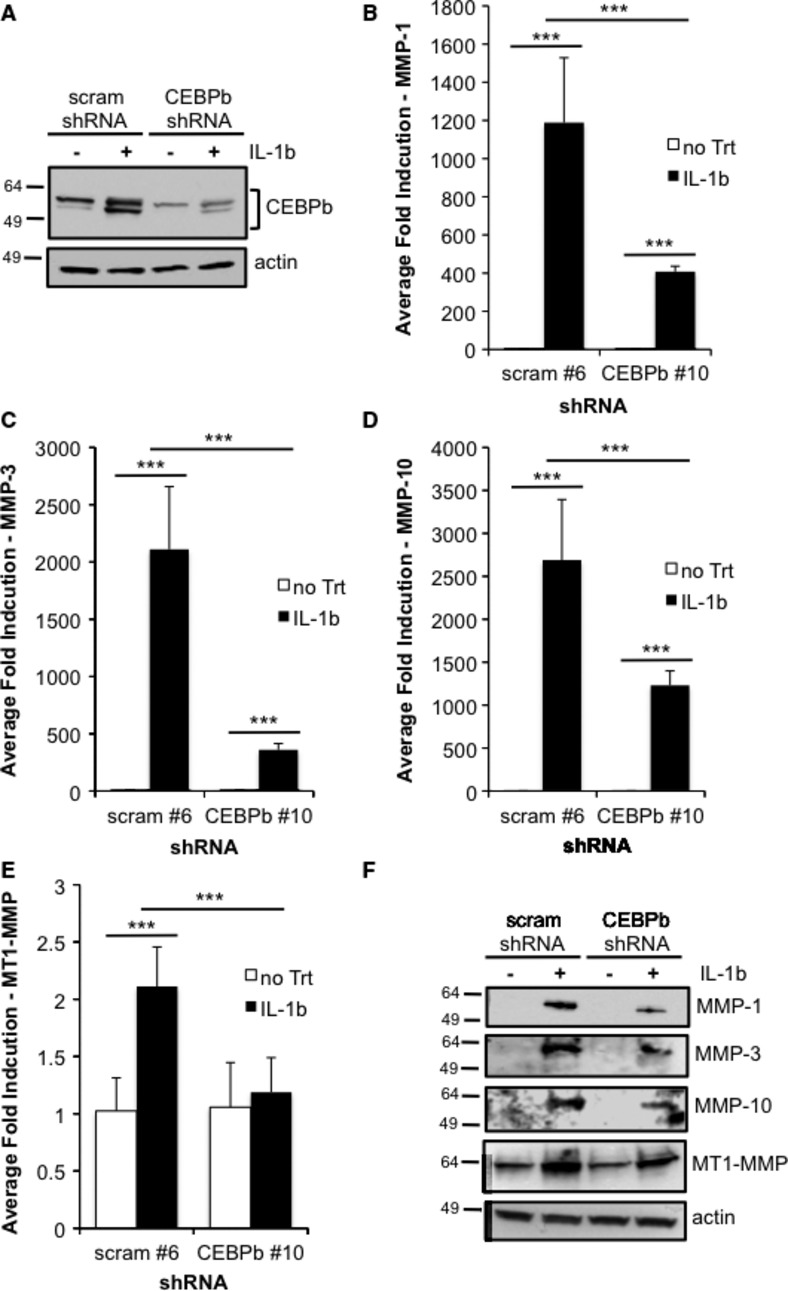
IL-1β-induced MMP expression is dependent on CEBPβ. Stable lines of 786-0 cells expressing either a scrambled shRNA or a CEBPβ-targeting shRNA were generated. Subconfluent cultures of individual clonal stable lines were treated overnight in serum-free media in the presence or absence of 1 ng/mL IL-1β. The following day, total RNA was harvested, and gene expression was analyzed by real-time PCR for MMP-1 (A), MMP-3 (B), MMP-10 (C), and MT1-MMP (D). Samples were performed in triplicate and experiments repeated twice. Data represent MMP gene expression normalized to GAPDH and calculated as average fold induction of untreated samples; *P* < 0.0001. (E) Stable polyclonal lines were treated overnight in serum-free media in the presence or absence of 1 ng/mL IL-1β. The following day, total protein was harvested, and media from the cultures were subjected to TCA precipitation for protein analysis of the secreted MMPs. Protein expression analyses of total CEBPβ, MMP-1, -3, -10, MT1-MMP, and actin were performed using Western blotting.

Our data thus far suggest that IL-1β-induced collagen invasion is mediated through the induction of MMP expression and the subsequent collagenolytic events that result from their activity. We next asked whether or not IL-1β-induced MMP expression was dependent on IL-1β-regulated CEBPβ expression. To this end, we generated stable cell lines that expressed either a scrambled shRNA or an shRNA specifically targeting CEBPβ. Stable expression of this shRNA significantly reduced basal and IL-1β-induced CEBPβ expression in the 786-0 cells (Fig. 5A). Knockdown of CEBPβ blocked IL-1β-induced MMP-1, MMP-3, and MMP-10 expression at both the mRNA and protein levels (Fig. 5B–D,F) with no effect on basal levels (data not shown). Although the high constitutive expression of MT1-MMP mRNA and protein were modestly induced by IL-1β compared to the other MMPs, this induction was also dependent on CEBPβ (Fig. 5E and F). These data demonstrate that CEBPβ is required for IL-1β induction of MMP expression in RCC cells.

### CEBPβ mediates IL-1β-induced RCC tumor cell invasion

**Figure 6:**
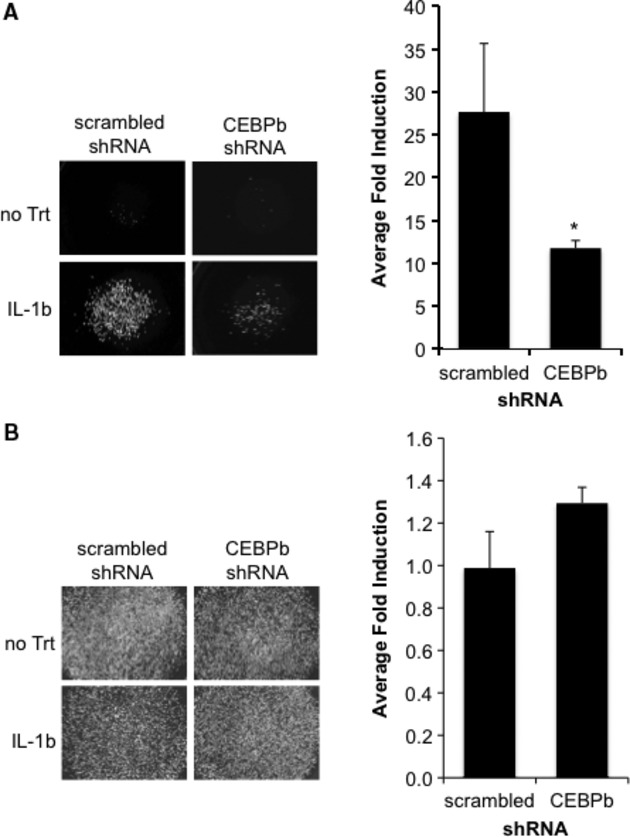
IL-1β-mediated collagen invasion of RCC cells is dependent on CEBPβ. Stable scrambled or CEBPβ shRNA 786-0 cells were treated overnight in serum-free media either alone or containing 1 ng/mL IL-1β. The next day, cells were harvested for the invasion assay (A) or migration assay (B) as described in the Methods and were allowed to invade or migrate for 24 h. Values are presented as average fold induction of untreated cells; *P* < 0.05.

Since we demonstrated that IL-1β-induced collagen invasion of RCC cells was dependent on MMP enzymatic activity (Fig. 1E) and that IL-1β-induced MMP expression was dependent on CEBPβ (Fig. 5), we next asked whether or not CEBPβ was required for IL-1β-mediated RCC tumor cell invasion. The stable scrambled shRNA and CEBPβ shRNA-expressing cell lines were treated overnight in serum-free media in the presence or absence of 1 ng/mL IL-1β before being seeded on top of type I collagen-coated transwells, as in Figure 1. CEBPβ knockdown significantly decreased IL-1β-induced tumor cell invasion (Fig. 6A). Importantly, CEBPβ knockdown had no effect on the migratory behavior of these cells at 24 h (Fig. 6B), suggesting that the IL-1β/CEBPβ/MMP axis specifically enables the cells to modify the collagen barrier present in the invasion assay. These studies demonstrate an important role for CEBPβ in mediating IL-1β-induced RCC tumor cell invasion.

## Discussion

Despite advances in our understanding of kidney tumor biology, metastatic RCC remains difficult to treat. Contributing to the low survival rate of advanced RCC patients is the fact that this cancer is refractory to chemotherapy and radiation. For years, the standard of care was immunotherapy in the form of high doses of interleukin-2 (IL-2) and/or interferon-α (INF-α). Unfortunately, patients suffered from acute toxicity, and the complete response rate was low [27]. Recently, molecularly targeted therapies, such as VEGF signaling inhibitors (sorafenib, sunitinib, bevacizumab) and mTOR inhibitors (everolimus, temsirolimus) have become FDA-approved as front-line therapy for RCC patients [28]. Although the adverse effects of these treatments are, in general, better tolerated compared to immunotherapy, tumors often develop resistance to these drugs. Furthermore, while the overall survival and progression-free survival rates of targeted therapies show improvement over cytokine therapy, curative rates are not superior [28]. Therefore, identifying new molecular targets in the biology of this cancer is important for the continued development of effective, well tolerated therapies.

In this report, we investigated the role of the inflammatory cytokine, IL-1β, in RCC tumor cell invasiveness as a key characteristic of a tumor cell's progression to metastatic disease. Our results indicate that IL-1β is sufficient to induce tumor cell invasion of RCC cells (Fig. 1). Moreover, IL-1β potently induced the expression of MMP-1, MMP-3, and MMP-10 and mildly induced the expression of MT1-MMP (Fig. 2). Of note, high levels of MMP-10 correlate with advanced stages of RCC, and MMP-10 is a poor prognostic factor for survival [29]. Expression of MMP-1, MMP-3, MMP-10 was coordinately upregulated by IL-1β, with mRNA expression detected as early as 2 h posttreatment (Fig. 3). It is possible that these MMPs are upregulated in an early response to IL-1β stimulation via direct interaction of CEBPβ at the promoters of these genes. In support of this hypothesis, we previously characterized a CEBPβ binding site at -2921 of the MMP-1 promoter and identified putative CEBPβ binding sites in the promoters of MMP-3 and MMP-10 [15]. In addition, our data show that IL-1β induced both CEBPβ expression and phosphorylation at threonine 235, an event required for CEBPβ transcriptional activity (Fig. 4; [14, 25]). Importantly, shRNA-mediated knockdown of CEBPβ reduced IL-1β-induced MMP-1, MMP-3, MMP-10, and MT1-MMP expression, thereby identifying these MMPs as downstream targets of CEBPβ (Fig. 5).

In contrast to the secreted MMPs, IL-1β induction of MT1-MMP was modest (Figs 2 and 3). This mild level of induction is likely due to the constitutive expression of MT1-MMP already present in these cells resulting from the lack of VHL expression and activation of HIF-2α [16]. In addition, MT1-MMP mRNA was not induced by IL-1β until 24 h, whereas the mRNAs of the other MMPs were induced as early as 2 h poststimulation (Fig. 3). These results suggest that unlike MMP-1, MMP-3, MMP-10, MT1-MMP may not be directly activated by CEBPβ, but activated in response to a downstream target of CEBPβ. One such candidate is IL-6, which is a well-described transcriptional target of CEBPβ [30]. In support of this hypothesis, others have reported MT1-MMP as a downstream target of IL-6 signaling [31]. In addition, IL-6 was potently induced by IL-1β in a CEBPβ-dependent manner by as early as 4 h posttreatment in our experiments (data not shown).

Using RNA interference, we next demonstrated that IL-1β-induced RCC tumor cell invasion required CEBPβ (Fig. 6). To our knowledge, this is the first report to demonstrate a direct role for CEBPβ in RCC tumor cell invasion. These data identify CEBPβ as an important mediator of the response of tumor epithelial cells to an inflammatory microenvironment. In agreement, others have reported a direct correlation between increased CEBPβ activity in RCC patient tumors and the presence of invasive disease [26]. Together, these findings provide rationale for designing RCC therapies that target the expression and/or activation of CEBPβ.

The role for inflammation in the progression of RCC is implicated by several clinical observations. For example, high serum C-reactive protein (CRP), an acute phase inflammatory protein, is a poor prognostic indicator for RCC [32–34]. High serum levels of the proinflammatory cytokines, IL-1β, IL-6, and TNFα, are also associated with advanced disease [10]. In addition, the presence of TAMs in RCC tumors is considered a poor prognostic indicator [8, 9], and inflammatory paraneoplastic syndromes are often associated with advanced RCC [35]. The reported serum concentration of IL-1β in metastatic RCC patients is approximately 500 ng/mL [10]. In our assays, we measured a dramatic increase in CEBPβ and MMP expression in response to concentrations of IL-1β as low as 0.1 ng/mL. Although in vitro and in vivo concentrations of IL-1β cannot be directly compared, it is interesting to note the responsiveness of the RCC cells to low IL-1β concentrations, which are far below that which is detected in patients. We postulate that the proinflammatory milieu expressed by TAMs in the tumor microenvironment has direct effects on the gene expression profiles of RCC tumor cells, thereby contributing to their progression to a metastatic phenotype. In summary, our results implicate the IL-1β/CEBPβ/MMP signaling pathway as a major mediator of RCC tumor cell invasion, and this pathway should be considered in the design of preclinical models of RCC to test its target-ability as an anti-metastatic therapy.
